# The Deaf Child: A Manual for Teachers and School Doctors

**Published:** 1912-09

**Authors:** 


**The Deaf Child: a Manual for Teachers and School Doctors.** By JAMES KERR LOVE, M.D. Pp. vi, 192. Bristol: John Wright & Sons Ltd. 1911.

Much of this book is a reasoned appeal for a more scientific application of existing educational methods in the training of the deaf child and a clinical basis is laid down with that purpose in view. The art of teaching the deaf is not described in detail, but the basic principles upon which that art is founded are considered. The unreasonableness of attempting to teach all deaf children in institutions, whether by the "oral," the "manual," or the "combined method," regardless of the degree of deafness, the mental attributes, and the physical perfection of the child, is clearly shown. The author's attitude may be stated in his own words, quoted from p. 124: "I assert that further progress in the education of the deaf-mute depends not on the study of methods of education, but on a study of the deaf themselves, a study which will give a scientific classification, and which will enable existing methods to be applied with greater efficiency."

There are chapters on the history of the education of the deaf, the physiology of hearing, the causes and estimation of deafness, and the operation of the language centres of the brain in normal and abnormal children.

In another chapter the author describes in brief the various institutions for teaching the deaf which he has visited at home and abroad, and his visitations of this kind have been very numerous and thorough.

Examples are given of the wonderful attainments possible for the well-trained deaf-mute. It is shown that the deaf child, at the end of his school career, may be turned out a capable and self-supporting citizen.

We advise all school doctors and teachers of the deaf to read this very thoughtful and suggestive manual.

